# Studies on the Interaction between Three Small Flavonoid Molecules and Bovine Lactoferrin

**DOI:** 10.1155/2018/7523165

**Published:** 2018-09-25

**Authors:** Junyi Huang, Zhenmin Liu, Qiaorong Ma, Ziyu He, Zhidian Niu, Mengying Zhang, Liu Pan, Xiaosheng Qu, Jun Yu, Bing Niu

**Affiliations:** ^1^State Key Laboratory of Dairy Biotechnology, Bright Dairy & Food Co. Ltd., Shanghai, 200436, China; ^2^Shanghai Key Laboratory of Bio-Energy Crops, School of Life Sciences, Shanghai University, 200444, China; ^3^Clinical Laboratory, Affiliated Minzu Hospital of Guangxi Medical University, Nanning, 530001, China; ^4^National Engineering Laboratory of Southwest Endangered Medicinal Resources Development, Guangxi Botanical Garden of Medicinal Plants, 530023 Nanning, China; ^5^Department of Ophthalmology, Xinhua Hospital, Shanghai Jiao Tong University School of Medicine, 200092, China

## Abstract

The interaction between three flavonoids, i.e., Luteolin (LTL), Quercetin (QCT), and Naringenin (NGN) and bovine lactoferrin (BLF) at pH 7.4 was investigated by fluorescence quenching spectra, synchronous fluorescence spectra, and UV-visible absorption spectra. The results indicate the fluorescence of BLF quenched by Luteolin (LTL), Quercetin (QCT), and Naringenin (NGN) via static quenching. The main force between QCT and LTL with BLF was van der Waals interactions and hydrogen bonds. Electrostatic interactions played a major role in the binding process of interaction between NGN and BLF. Synchronous fluorescence was used to study the conformational changes of BLF. The values of binding constant (Ka) and number of binding sites (n) at different temperatures (300K, 305K, 310K) were also calculated, respectively. The results of corresponding thermodynamic parameters as well as binding distance between BLF and LTL, QCT, or GNG were obtained. These results implied that Luteolin (LTL), Quercetin (QCT), and Naringenin (NGN) could provide important guides for compound quantity (e.g., medicine dosage) and the design of new compounds (or drugs).

## 1. Introduction

Flavonoids are phytochemicals found in fruit, vegetables, nuts, seeds, stems, and flowers as well as tea, wine, propolis, and honey, which are known to be responsible for colors of many flowers and fruits and protect the plants against pathogens, insects, and UV B radiation [1]. The widespread distribution of flavonoids means that many animals, including humans, ingest significant quantities of flavonoids in their diet. Flavonoids possess many useful properties, including anti-inflammatory activity, oestrogenic activity, enzyme inhibition, antimicrobial activity [2], antiallergenic activity, antioxidant activity, vascular activity, and cytotoxic and antitumor activity [3]. Increasingly, flavonoids are the subject of medical research.

Lactoferrin (LF) is a nonhemic iron-binding glycoprotein found in secretions from exocrine glands, including tears, saliva, semen, bile, and specifically granules of neutrophils [4]. LF is a multifunctional protein involved in many physiological functions, including anti-inflammatory activity, iron transport function, broad-spectrum antibacterial action, regulation of cellular growth and differentiation, and anticancer effect. LF is mainly secreted by the lacrimal gland tear, a small part from the accessory lacrimal gland. The rich content of LF is found in tears, and tear lysozyme, prealbumin, a small amount of secretory immunoglobulin, and growth factors constitute the main ingredients in tear protein. LF is the main defense force to protect the eye from invasion which can cause various ocular surface diseases. In view of its important physiological functions, many drug molecules, such as lomefloxacin [5], tosufloxacin [6], oleic acid [7], and polyphenon [8], have been reported to interact with LF.

Protein binding played a potential role in distribution, excretion, and therapeutic effects, and it has been considered as one of the most important physical and chemical characteristics of drugs. The study of the binding of small molecules to protein is an essential and fundamental importance [9]. A new trend in protein-small molecular interaction research is the use of different spectroscopic analyses combined with computational methods (molecular docking) to obtain the mode of interaction of the binding partners [10–13]. Isothermal titration calorimetry (ITC) was also performed [9]. Anbazhagan investigated differential interactions of artemisinin and its derivatives with serum albumin by fluorescence measurements, stopped-flow spectroscopy, and molecular modeling [14]. The UV-visible absorbance spectroscopy and infrared spectroscopy (IR) have been used to research the binding properties of isomeric drugs to BLF [15]. Circular dichroism spectra (CD), a precise method to confirm the changes of proteins conformation, yield information about the three-dimensional structure of protein binding site reduce by small molecules or drugs [16, 17]. Besides the above methods, Moosavi applied cyclic voltammetric for the molten globule states of cytochrome c induced by n-alkyl sulfates [18].

As a bioactive compound with antibacterial and antioxidant activities, flavonoid micromolecules may possibly have an impact on activity of BLF in inflammatory reactions. Zhang et al. [19] study the biological implication of the interaction between fibrinogen and resveratrol and observed that fibrinogen strengthened the stability of resveratrol. Li [20] studied interactions between 3 flavonoid compounds and *α*-amylase, and the results showed that the 3 flavonoid compounds are effective inhibitors of *α*-amylase. Therefore, in our study, the interaction between Luteolin (LTL), Quercetin (QCT), Naringenin (NGN), and BLF was investigated with UV-visible spectroscopy and fluorescence spectroscopy.

## 2. Materials and Methods

### 2.1. Samples and Reagents

Luteolin (LTL), Quercetin (QCT), and Naringenin (NGN) were purchased from Sigma-Aldrich Chemical Co. Ltd. Bovine LF (98%, purity) was purchased from the Shanghai Huicheng Biotech Co. Ltd. All other chemicals were of analytical grade and used without further purification.

### 2.2. Sample Preparation

A bovine LF stock solution was made by dissolving the appropriate amount in amount of LF in 0.05 M phosphate buffer (pH 7.4). Solutions of Luteolin, Quercetin, and Naringenin were prepared daily by dissolving the appropriate amount in pure alcohol to obtain concentration 10 mM, respectively.

Samples were prepared by mixing BLF solutions and LTL, QCT, or NGN of varying proportions. The resulting ethanol concentration was approximately 1%, which had no appreciable effect on protein structure. All samples were kept at 277K before determination.

### 2.3. Procedures

#### 2.3.1. Fluorescence Measurement

Fluorescence measurements were run with a spectrofluorometer, Model LS-55 (PerkinElmer, USA), equipped with a thermostatic sample compartment, and connected with a circulating bath (Lauda, K-2R; Brinkmann Instruments, Westbury, NY, USA).

2.5 mL of 0.5 *μ*M BLF solution was put in 1.0 cm quartz cells which were titrated by successive additions of 2.5 mM LTL, QCT, or NGN solutions according to the concentrations of 0, 1, 2, 3, 4, 5, 6, 7, and 8 *μ*M, respectively. Fluorescence emission spectra of BLF were scanned from 300 to 500 nm with the excitation wavelength of 280 nm at 300 K, 305 K, and 310 K.

The band-widths of excitation and emission wavelength were set at 8 nm, and the scanning speed was 220 nm/min. The synchronous fluorescence spectra were determined in the continuous range of 200 to 500 nm with Δ*λ* at 15 and 60 nm.

In order to avoid the inner filter effects of protein and ligands, absorbance measurements were made at excitation and emission wavelengths of bovine LF. The fluorescence intensity was corrected using the following equation [11]:

(1) $$\begin{array}{c} F_{cor}=F_{obsd}e\frac{A_{280}+A_{345}}{2} \end{array}$$

where F_cor_ and F_obsd_ are the absorption-corrected fluorescence and the observed fluorescence, while A_280_ and A_344_ are the sums of the absorbance of protein and ligand at excitation and emission wavelengths, respectively. However, in the flavonoid-BLF ultraviolet absorption spectrum, LTL, QCT, or NGN does not exhibit any absorption at the excitation wavelength (280 nm) or at the emission wavelength (345 nm). So, no inner filter effects need to be considered [14].

#### 2.3.2. UV-Visible Absorption Measurement

Absorbance spectra were recorded on a VARIAN 100 UV-VIS spectrophotometer (Varian Australia Pty. Ltd., Australia).

2.5 mL of 1 *μ*M BLF solution in 1.0 cm quartz cells was titrated by successive additions of 2.5 mM LTL, QCT, or NGN solutions for concentrations of 0, 1, 2, 3, 4, 5, 6, 7, and 8 *μ*M, respectively, and their absorption spectra were recorded from 200 to 500 nm.

### 2.4. Molecular Docking

SYBYL X-2.0 software was used for molecular docking based on its Surflex-Dock module. The crystal structure of protein with the resolution of 2.6 Å was downloaded from the Protein Data Bank (PDB ID: 1M17). Protein was prepared using protein structure preparation module of the SYBYL X-2.0 software. All the water molecules and ligand were deleted, and hydrogen atoms were added to the crystal structure. In addition, the terminal-treatment of the protein was added charge. Small molecules were minimized at physiological pH7.4 with hydrogen atoms and charge by using Powell energy gradient method and the Gasteiger-Huckel system. The tautomeric form of the minimized inhibitor was free.

### 2.5. Statistical Analysis

All determinations were performed in triplicate and the mean values and standard deviations were analyzed by using SPSS 13.0 for Windows (SPSS Inc., IL, USA).

## 3. Results

### 3.1. Interactions between Flavonoids and BLF

**Figure 1:**
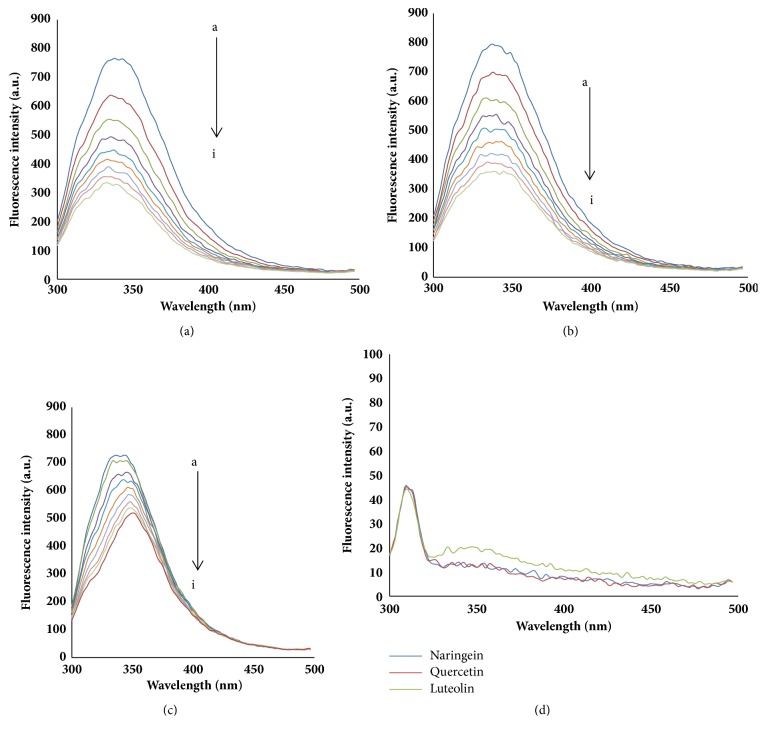
The fluorescence quenching spectra of BLF in the presence of different concentrations of three flavonoids. ((a) The fluorescence quenching spectra of BLF in the presence of different concentrations of LTL. (b) The fluorescence quenching spectra of BLF in the presence of different concentrations of QCT. (c) The fluorescence quenching spectra of BLF in the presence of different concentrations of NGN. C_BLF_=1.0 × 10^−6^mol/L, a-i: C_LTL_ = C_QCN_ = C_NGN_ =(0,1,2,3,3,5,6,7,8)×10^−6 ^mol·L^−1^, T=300K, pH=7.4, *λ*ex=280nm.)

As shown in Figure 1, BLF's fluorescence intensity of approximately 344 nm regularly decreased with different concentrations of the added flavonoids. Furthermore, the maximum wavelength of BLF had a hypochromatic shift from 344 to 331 nm, as shown in Figure 1(a), and from 344 to 338 nm, as shown in Figure 1(b), respectively. It has been shown that Trp creates a more hydrophobic environment [21]. However, the peak position had a redshift from 344 to 349 nm, as shown in Figure 1(c), implying that the polarity of the microenvironment around Trp increased after NGN was added to the BLF solution. The fluorescence intensity of BLF decreased with increasing flavonoid concentrations, which indicated that the BLF conformation may be changed and that intermolecular energy transfer occurred between BLF and flavonoid [11].

**Figure 2:**
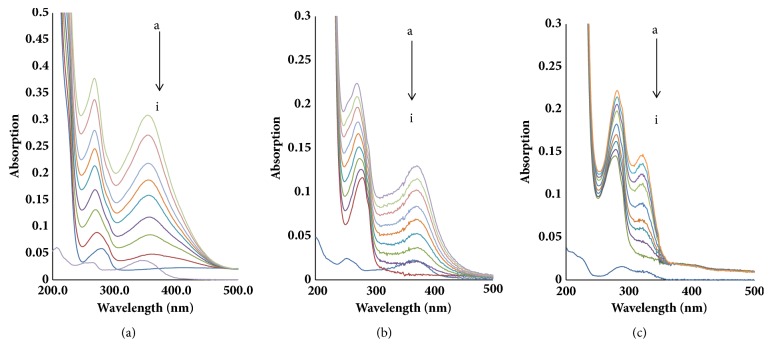
UV-visible spectra of BLF in the presence of three flavonoids. ((a) UV-visible spectra of BLF in the presence of LTL. (b) UV-visible spectra of BLF in the presence of QCT. (c) UV-visible spectra of BLF in the presence of NGN. C_BLF_=1.0 × 10^−6^mol/L, a-i: C_LTL_ = C_QCN_ = C_NGN_ =(0,1,2,3, 4,5,6,7,8)×10^−6 ^mol/L. j: 8 *μ*M flavonoid alone.)

The influence of the flavonoids on the UV-visible spectra of BLF is shown in Figure 2. BLF without addition exhibited a maximum absorption peak at 280 nm (curve j), but BLF showed a blueshift from 280 nm to 268 nm or from 280 nm to 270 nm with the addition of LTL or QCT, respectively, and a redshift from 280 nm to 283 nm with the addition of NGN (curves a-i). Simultaneously, the peak values gradually increased, suggesting the interactions between three flavonoids and BLF occurred, primarily induced by hydrogen bonding or hydrophobic interaction. Moreover, the addition of the flavonoids resulted in the presence of a new peak at 370 nm, which is the characteristic peak of flavonoids, and the flavonoids' absorption values increased with the increase in flavonoids concentration, which further confirmed the occurrence of interactions between three flavonoids and BLF.

### 3.2. The Fluorescence Quenching Mechanism

For experiments performed with large molar protein to drug ratios, it is hypothesized that each binding site is active in the binding drug, is identical, and acts independently. Given the validity of these assumptions, the fluorescence quenching behaviour can be analyzed using the Stern–Volmer and Lehrer equations for linear and nonlinear (hyperbolic) fits, respectively. Dynamic quenching was calculated according to Stern–Volmer equation [22]:

(2) $$\begin{array}{c} \frac{F_{0}}{F}=1+Kq\tau_{0}\left[\;Q\;\right]=1+Ksv\left[\;Q\;\right] \end{array}$$

where *F*_0_ and* F *are the relative fluorescence intensities of BLF at 345 nm in the absence and presence of a quencher,* Kq* is the quenching rate constant of the bimolecule, *τ*_0_ is the average lifetime of biomolecule without active constituents, [*Q*] is the concentration of active constituent, and* K*_*SV*_ stands for the Stern–Volmer dynamic quenching constant.

**Figure 3:**
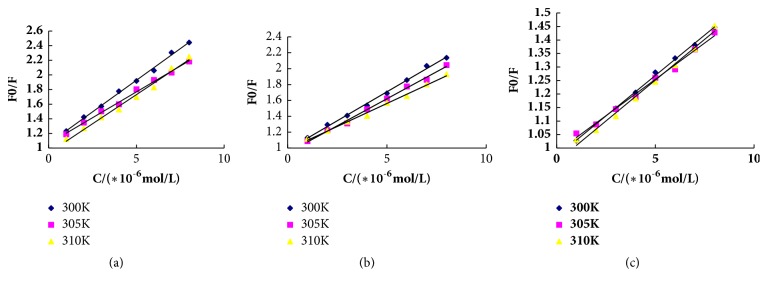
Stem-Volmer plots of three flavonoids-BLF systems at different temperatures. ((a) Stem-Volmer plots of LTL-BLF systems at different temperatures. (b) Stem-Volmer plots of QCT-BLF systems at different temperatures. (c) Stem-Volmer plots of NGN-BLF systems at different temperatures.)

To confirm the possible quenching mechanism of the three flavonoids binding to BLF, the dynamic quenching parameters,* Ksv* and* Kq,* were achieved from the experimental results using Stern–Volmer equation. The fluorescence lifetime of the biopolymer is 10^−8^ s. The linear plot of F_0_/F as a function of Quercetin concentration is given in Figure 3; the results are listed in Table 1. The maximum scatter collision quenching constant of various quenchers with the biopolymer is 2.00 × 10^10^ M^−1^ [23]. The values of* Kq* decreased with increased temperature and were greater than the limiting diffusion constant, which suggested that the possible quenching mechanism was a static quenching process accompanied with the formation of BLF–flavonoid complexes, and dynamic collision was negligible.

### 3.3. Binding Parameters

For the static quenching, the binding constant (*K*_*a*_) and the number of binding sites (*n*) can be calculated using the following equation [24]:

(3) $$\begin{array}{c} \mathrm{lg}\left[\;\frac{\left(F_{0}-F\right)}{F}\;\right]=\mathrm{lg}K_{a}+n\mathrm{lg}\left[\;Q\;\right] \end{array}$$

And, for n ≈1, (3) can be rewritten as follows [25]:

(4) $$\begin{array}{c} \frac{F0}{\left(F0-F\right)}=1+K_{a}^{-1}{\left[\;Q\;\right]}^{-1} \end{array}$$

The results are exhibited in Tables 1 and 2.

BLF interacts with LTL, QCT, and GNG to form 1:1 complexes. With increased temperature, the values of *K*_*a*_ obtained at the excitation wavelength of 280 nm decreased, which may indicate the formation of an unstable compound. The unstable compound would be partly decomposed with the increased temperature. Moreover, it has been reported that an equilibrium between monomeric and associated states may exist in solution due to self-association at high concentrations [26].

The binding constants have an order of magnitude of 10^4^ L·mol^−1^, which means that the binding strength is relatively high, indicating strong binding affinities. This is further evidence that the binding interaction between flavonoids and bovine lactoferrin occurs. The binding constants obtained for the BLF complexes are in the range of 1.79 × 10^4^ M^−1^ - 9.68 × 10^4^ M^−1^, and similar results were generally observed for BL-artemisinin complexes (1.6 × 10^4^ M^−1^ - 6.2 × 10^4^ M^−1^) [14]. These results indicated that LTL and QCN bound to HSA to a larger extent than to NGN, which signifies that the affinity of BLF to LTL or QCN was higher than that between BLF and QCN.

### 3.4. Thermodynamic Parameter and Nature of the Binding Force

There are some interaction forces between an active constituent and a biomacromolecule, such as hydrophobic forces, van der Waals interactions, electrostatic interactions, and hydrogen bonds. The signs and magnitudes of thermodynamic parameters that are calculated from the van 't Hoff equation account for the main forces maintaining protein stability [27]. From Table 3, it is observed that the negative sign for* △G* indicates the binding spontaneity of the three flavonoids with BLF (see (5)-(6)). According to the views of Timasheff, Ross, and Subramanian, from the model of the interaction between the drug and HSA, the interactions can be concluded: (1)* △H* > 0 and* △S* > 0, hydrophobic forces; (2)* △H* < 0 and* △S* < 0, van der Waals interactions and hydrogen bonds; and (3)* △H* < 0 and* △S* > 0, electrostatic interactions. Hence, the results showed that the main force between QCT and LTL with BLF is van der Waals interactions and hydrogen bonds, and electrostatic interactions played a major role in the binding process between BLF and NGN.

(5) ln⁡Ka=−△HRT+△SR

(6) △G=△H−T△S

### 3.5. Energy Transfer from BLF and Quercetin

According to Forster's nonradiative energy transfer theory [28], the energy can be transferred from the donor to the acceptor when a donor is emitted by fluorescence.

**Figure 4:**
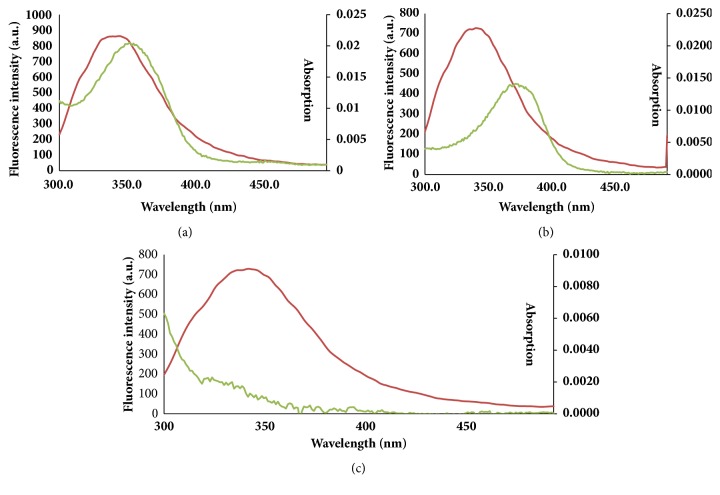
Overlap of absorption spectra of three flavonoids and fluorescence mission spectra of BLF. (a) Overlap of absorption spectra of LTL. (b) Overlap of absorption spectra of QCT. (c) Overlap of absorption spectra of NGN. C_BLF_ = C_LTL_ = C_QCN_ = C_NGN_ = 1.0 × 10^−6^mol·L^−1^.

The fluorescence quenching of BLF after binding to Quercetin indicated that the transfer of energy between Quercetin and BLF occurred. Spectroscopy of the donor is malformed due to this interaction. The related parameters, including energy transfer efficiency* E*, the distance (*r*), and the critical energy transfer distance (*R*_0_), are calculated by the following equations:

(7) E=R06R06+r06

(8) E=1−FF0

(9) R06=8.8×10−25 K2N−4ΦJ

(10) J=∑Fλ·ελ·λ4·Δλ∑Fλ·Δλ

where *R*_0_ is the critical distance when the transfer efficiency is 50%, *K*^2^ stands for the spatial orientation factor of the dipole,* N* is the refractive index of the medium,* F *stands for the fluorescence quantum yield of the donor, and* J* stands for the overlap integral of the fluorescence emission spectrum of the donor and the absorption spectrum of the acceptor.* F(λ)* stands for the fluorescence intensity of the donor and* ε(λ)* is the molar absorptivity of the acceptor when the wavelength is *λ*. In the experiment, *K*^2^ is 2/3,* N* is 1.36, and* Φ* is supposed to be the same as the fluorescence quantum yield of tryptophan (0.15). The concentration of flavonoid is very low, so its absorbance values are low. The calculated results are shown in Figure 4 and Table 4. The distance (r) between Quercetin and BLF was less than 7 nm, which verified the presence of nonradiation energy transfer between Quercetin and BLF [29].

### 3.6. Effect of Flavonoids on BLF Conformation

**Figure 5:**
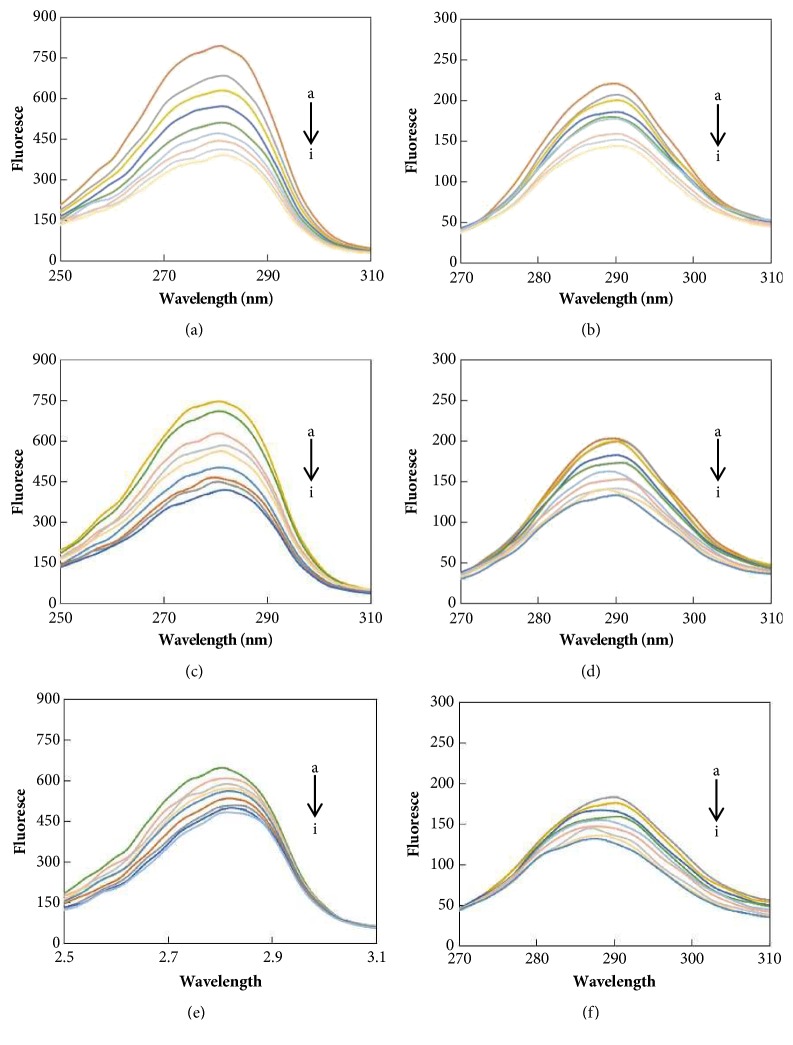
Effect of the drug on the synchronous fluorescence spectrum of BLF. (a) Δ*λ* = 15 nm UA-BLF; (b) Δ*λ* = 60 nm OA-BFL; (c) Δ*λ* = 15 nm UA-BFL; (d) Δ*λ* = 60 nm OA-BFL; (e) Δ*λ* = 15 nm UA-BFL; (f) Δ*λ* = 60 nm OA-BFL. C_BLF_=1 *μ*M, a-i: C_LTL_ = C_QCN_ = C_NGN_ =(0,1,2,3,3,5,6,7,8)×10^−6 ^mol·L^−1^.

Synchronous fluorescence of the flavonoid-BLF systems was detected to investigate the microenvironment change of amino acid residues, which is related to a shift in the maximum emission wavelength, and the results are shown in Figure 5. When Δ*λ* (Δ*λ*=*λ*emission-*λ*excitation) is 15 or 60 nm, the synchronous fluorescence provides the characteristic information of tyrosine residues or tryptophan residues, respectively, due to the changes of the polarity in the microenvironment [30].

Obviously, the conformations of BLF were changed with the addition of LTL, QCT, and NGN. A redshift effect suggests that the polarity around the tyrosine residues increased, and the hydrophobicity decreased; a hypochromatic shift effect suggests that the polarity decreased around the tryptophan residues and increased in the hydrophobicity.

### 3.7. Analysis of Molecular Docking

**Figure 6:**
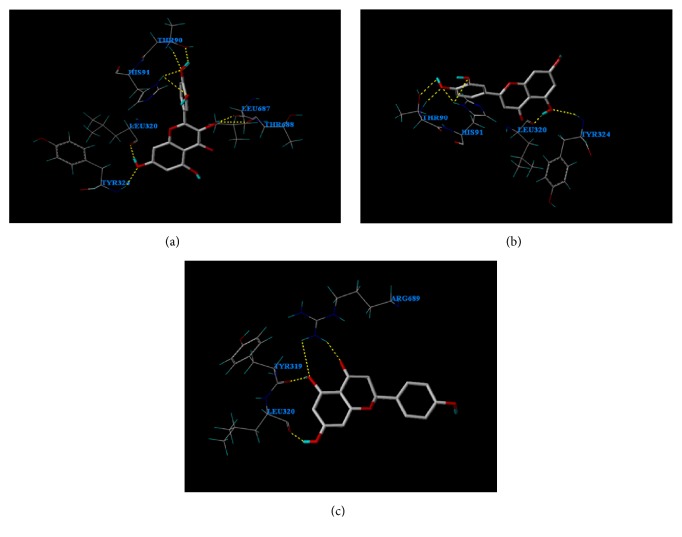
Molecule docking. (Molecule docking between LTL and BLF. Molecule docking between QCT and BLF. Molecule docking between NGN and BLF.)

Molecular docking of the three flavonoids with BLF was studied using SYBYL-X-2.0.  Figure 6 shows that Luteolin (LTL) and Quercetin (QCT) bind to BLF via hydrogen bonds. LTL interacts with THR90 and HIS91 with two hydrogen bonds, respectively; and it also binds to BLF at LEU320, TYR324, LEU687, and THR688 with one hydrogen bond, respectively. In this study, there are a total of eight hydrogen bonds when LTL interacts with BLF. QCT forms two hydrogen bonds with THR90 and HIS91, respectively. In addition, QCT binds to BLF at LEU320 and TYR324 with one hydrogen bond, respectively. Hence, it is suggested that hydrogen bonds contribute the most to LTL and QCT interaction with BLF. NGN formed two hydrogen bonds with ARG689 and formed a single hydrogen bond with TYR319 and LEU320, respectively. Obviously, it is possible that other possible forces, such as hydrophobic interactions, are essential for the binding between NGN and BLF. The flavone nucleus is the main hydrophobic group, which interacts with BLF via a hydrophobic force. The docking results agreed well with the aforementioned spectroscopy results.

## 4. Conclusion

In this work, we studied the interactions between BLF and 3 flavonoid compounds using the spectroscopy methods. The results revealed that LTL, QCT, and NGN interact with BLF to form new complexes, which leads to the static quenching of the fluorescence of BLF and nonradiation energy transfer. In these results, both van der Waals interactions and hydrogen bonds played key roles in the binding process of Lut and Que with BLF; however, the main forces between BLF and NGN are electrostatic interactions. The binding affinity of the flavonoids to BLF was the greatest for LTL followed by QCT and was least for NGN. The results of the UV-vis spectra and the synchronous fluorescence analysis showed that the formation of flavonoid-BLF complexes induced changes of different degrees in the protein structure.

The above studies showed that the following relationships may exist for the structure and binding interactions of the three flavonoids with BLF: (1) hydroxyl groups at the 5, 7 position of ring A in the three flavonoids were key to determining the groups, (2) the increase of phenolic hydroxyls in the B ring of the flavonoids was favourable for drug binding to HSA, and (3) C4'-OH enhanced the binding affinity obviously, but C3-OH weakened the affinity.

The binding properties of flavonoids with proteins can help to characterize the biological process of the compound. The results of ligand protein binding can provide important guides for compound quantity (e.g., medicine dosage) and the design of new compounds (or drugs).

## Figures and Tables

**Table 1:** Stern–Volmer equation of fluorescence quenching of BLF due to its interaction with LTL, QCT, or NGN and values of Ksv, Kq, and Runder temperatures of 300 K, 305 K, and 310 K. All titrations were performed in triplicate.

	*T* (K)	*Ksv* (×10^4^ M^−1^)	*Kq* (×10^12^ M^−1^s^−1^)	*R* ^a^ (correlation coefficien)	*SD* ^b^
Luteolin	300	10.08	10.08	0.997	0.04992
	305	9.14	9.14	0.999	0.01584
	310	8.94	8.94	0.997	0.03678
Quercetin	300	9.81	9.81	0.998	0.02643
	305	8.58	8.58	0.998	0.03384
	310	8.00	8.00	0.997	0.04759
Naringein	300	3.67	3.67	0.994	0.06257
	305	3.14	3.14	0.997	0.04757
	310	3.06	3.06	0.999	0.0236

^a^ *R *is the correlation coefficient for the *Ksv* values.

^b^  SD is the standard deviation for the *Ksv* values.

**Table 2:** Binding constant *K*_*a*_ and number of binding sites of the interaction of LTL, QCT, or NGN with BLF.

	*T* (K)	*Ka* (×10^4^M^−1^)	*n* ^e^	*R* ^c^	*SD* ^d^
Luteolin	300	8.889	1.052	0.997	0.06442
	305	8.410	1.020	0.999	0.03862
	310	7.257	1.079	0.998	0.03582
Quercetin	300	9.689	1.034	0.998	0.04577
	305	7.215	1.050	0.998	0.03874
	310	5.347	1.105	0.997	0.05829
Naringein	300	3.05	1.076	0.999	0.04278
	305	2.35	1.166	0.971	0.08442
	310	1.79	1.078	0.992	0.06793

^c^ *R is* the correlation coefficient for the *K*_*a*_ values.

^d^  SD is the standard deviation for the *K*_*a*_ values.

^e^  The binding site (*n*) approximated to 1.

**Table 3:** Thermodynamic parameters of the interaction of LTL, QCT, or NGN with BLF.

	*K*_*a*_ × 10^4^ at 310 K (M^−1^)	*△H* (kJ mol^−1^)	*△S* (J mol^−1^ K^−1^)	*△G* at 310 K (kJ mol^−1^)
Luteolin	7.257	-15.30	+41.79	-27.84
Quercetin	5.347	-31.48	-10.13	-28.42
Naringenin	1.79	3.57	74.60	-26.86

**Table 4:** Binding distances of the interaction of BLF with LTL, QCT, or NGN.

compound	J (×10^−17^cm^3^ l mol^−1^)	R_0_(nm)	E	R (nm)
Quercetin	5.45	2.59	0.112	3.66
Naringenin	3.61	2.11	0.042	3.56
Luteolin	6.36	2.80	0.034	4.89

## Data Availability

The data used to support the findings of this study are available from the corresponding author upon request.
